# Midostaurin preferentially attenuates proliferation of triple-negative breast cancer cell lines through inhibition of Aurora kinase family

**DOI:** 10.1186/s12929-015-0150-2

**Published:** 2015-07-04

**Authors:** Masaaki Kawai, Akio Nakashima, Shinji Kamada, Ushio Kikkawa

**Affiliations:** Biosignal Research Center, Kobe University, Kobe, 657-8501 Japan; Department of Biology, Graduate School of Science, Kobe University, Kobe, 657-8501 Japan; Department of Bioresource Science, Graduate School of Agricultural Science, Kobe University, Kobe, 657-8501 Japan; Central Research Laboratories, Sysmex Corporation, Kobe, 651-2271 Japan; Present Address: Research Center for Environmental Genomics, Kobe University, Kobe, 657-8501 Japan

**Keywords:** Triple-negative breast cancer, Midostaurin, Clustering analysis, Aurora kinase

## Abstract

**Background:**

Breast cancer is classified into three subtypes by the expression of biomarker receptors such as hormone receptors and human epidermal growth factor receptor 2. Triple-negative breast cancer (TNBC) expresses none of these receptors and has an aggressive phenotype with a poor prognosis, which is insensitive to the drugs that target the hormone receptors and human epidermal growth factor receptor 2. It is, thus, required to develop an effective therapeutic reagent to treat TNBC.

**Results:**

The study using a panel of 19 breast cancer cell lines revealed that midostaurin, a multi-target protein kinase inhibitor, suppresses preferentially the growth of TNBC cells comparing with non-TNBC cells. Clustering analysis of the drug activity data for the panel of cancer cell lines predicted that midostaurin shares the target with Aurora kinase inhibitors. Following studies indicated that midostaurin attenuates the phosphorylation reaction mediated by Aurora kinase in the cells and directly inhibits this protein kinase *in vitro*, and that this reagent induces apoptosis accompanying accumulation of 4N and 8N DNA cells in TNBC cells.

**Conclusion:**

Midostaurin suppresses the proliferation of TNBC cells among the breast cancer cell lines presumably through the inhibition of the Aurora kinase family. The precise study of midostaurin on cell growth will contribute to the development of the drug for the treatment of TNBC.

**Electronic supplementary material:**

The online version of this article (doi:10.1186/s12929-015-0150-2) contains supplementary material, which is available to authorized users.

## Background

The human genome encodes more than 500 protein kinases, which are divided into two major groups recognizing the hydroxyl side chains of serine/threonine and tyrosine, respectively [1–3]. It is well known that the protein kinases are involved in various aspects of fundamental cellular functions including growth and proliferation: perturbations of protein kinases caused by mutation and overexpression have been implicated in carcinogenesis and its development, and aberrantly activated protein kinases are regarded as potential therapeutic targets for cancers [4]. For example, the screening for the inhibitors against the constitutively activated tyrosine kinase BCR-ABL, which is generated by chromosomal translocation and gene fusion in chronic myeloid leukemia, identified the small molecule inhibitor imatinib as a molecular-targeted anticancer drug [5]. Midostaurin, also known as PKC412, is a protein kinase inhibitor originally developed against PKC, a serine/threonine protein kinase family [6]. It has later been shown that this compound inhibits not only a series of serine/threonine protein kinases including the PKC family but also tyrosine protein kinases [7]. FLT-3 (FMS-like tyrosine kinase-3) receptor kinase is one of the targets of midostaurin, and its mutations are frequently found in acute myeloid leukemia (AML), which result in the constitutive activation of FLT-3 and induce cell proliferation [8]. Thus, midostaurin has been developed as a drug for the treatment of AML. Midostaurin, on the other hand, is effective on AML and other cell lines derived from solid tumors without aberration in FLT-3 [6, 9]. As midostaurin is a multi-target protein kinase inhibitor having a broad inhibition spectrum [7], it is suggested that midostaurin shows its anti-cancer effect against the target other than FLT-3. Little is, however, known about the molecular mechanism for the midostaurin as a multi-target anti-cancer reagent.

Breast cancer is one of the most common malignancies in women, nowadays, which is classified into the subtypes according to the expression of biomarkers: the hormone and growth factor receptors. They include the subtype expressing human epidermal growth factor receptor 2 (HER2), the subtype expressing endocrine receptors such as estrogen and progesterone receptors without expression of HER2 (ER+), and the subtype with none of these receptors called triple-negative breast cancer (TNBC) [10]. TNBC, highly overlapping with the basal-like subgroup defined by microarray analysis [11], has an aggressive phenotype with a relatively poor prognosis. There is no specific drug for TNBC, although hormone therapy is established for the treatment of ER+ breast cancer, and lapatinib, a small molecule inhibitor for human epidermal growth factor receptor 2, is employed for HER2 breast cancer, which also inhibits epidermal growth factor (EGF) receptor [10, 11]. It is, thus, required to develop an effective drug to treat this breast cancer subtype.

We examined the effect of several protein kinase inhibitors on a panel of 19 breast cancer cell lines covering these three subtypes, and found that midostaurin preferentially suppresses cell growth of the TNBC cell lines. The breast cancer cells do not express FLT-3 [12], and thus it is of interest to identify the target of midostaurin in the TNBC cell lines. On the other hand, the clustering analysis of the drug activity data for a panel of cancer cell lines has been widely used as a tool for the development of anti-cancer drugs [13]. As drugs sharing the target molecule(s) show similar growth inhibition patterns among the cancer cell lines, it is possible to line up the tested drug along with the compounds having known targets [13]. The combination of the results of midostaurin obtained herein and data of the previous study [14] followed by the clustering analysis predicted that midostaurin shares the target with Aurora kinase inhibitors in breast cancer cell lines.

Aurora kinase is a family of serine/threonine protein kinases consisting of three members of A, B, and C, which have crucial roles for cell division by controlling chromatid segregation and cytokinesis [15]. Aurora kinases A and B are found in various tissues, but the expression of Aurora kinase C is restricted in testis and its overexpression has been reported in certain cancer cells [15]. Dysregulation of Aurora kinases A and B has been implicated in various cancers including AML [16] and breast cancer [17], and thus Aurora kinases A and B have been regarded to be possible targets of the cancer therapy [15]. Therefore, the effect of midostaurin on these Aurora kinases was studied in the breast cancer cell lines.

## Methods

### Cell lines and culture conditions

Breast cancer cell lines used in this study were purchased from American Type Culture Collection (ATCC, Rockville, Maryland): ER+ includes HCC1428, MCF7, and ZR-75-1; HER2 includes AU565, BT-474, HCC1419, HCC1954, MDA-MB-453, SK-BR-3, and ZR-75-30; TNBC includes BT-20, BT-549, HCC1806, HS578T, MDA-MB-157, MDA-MB-231, MDA-MB-435S, MDA-MB-436, and MDA-MB-468 [18–20]. The cells were cultured under the conditions described in Additional file 1 with fetal bovine serum (FBS) purchased from Thermo Fisher Scientific (Waltham, Massachusetts).

### Inhibitors

Midostaurin and lapatinib were purchased from LC Laboratories (Woburn, Massachusetts). VX-680 was from Symansis (Temecula, California). These inhibitors were dissolved in dimethyl sulfoxide (DMSO) at 10 mM and stored at −20 °C until use. The final concentration of DMSO in the culture medium and kinase assay mixture was 0.1 %.

### Antibodies

Antibodies were obtained from commercial sources. Antibodies against Aurora kinase A (#12100), p-Aurora A Thr288/Aurora B Thr 232 (#2914), EGF receptor (EGFR) (#2232), p-EGFR Tyr1068 (#3777), p-GSK-3β Ser9 (#9336), p-Akt Ser473 (#4060), Akt (#4691), p-Erk Thr202/Thr204 (#4370), Erk 1/2 (#9102), poly(ADP-ribose) polymerase (PARP) (#9542), PKC-α (#2056), PKC-δ (#2058), PKC-ε (#2683), and p-Serine PKC substrates (#6967) were purchased from Cell Signaling Technology (Beverly, Massachusetts). Antibody against Aurora B (#ab45145) was purchased from Abcam (Cambridge, UK). Antibody against GSK-3β (#610201) was purchased from BD Bioscience (San Jose, California). Antibodies against Histone H3 (#sc-10809), PKC-βII (#sc-210) and γ-tubulin (#sc-51715) were purchased from Santa Cruz Biotechnology (Santa Cruz, California). Antibody against glyceraldehyde-3-phosphate dehydrogenase (GAPDH) (#2275-pc-100) was purchased from Trevigen (Gaithersburg, Maryland). The antibody against p-Histone H3 Ser10 (#06-570) was purchased from Millipore (Billerica, Massachusetts). Horseradish peroxidase-conjugated secondary antibodies against rabbit and mouse IgG were purchased from Dako (Glostrup, Denmark).

### Cell viability assay

Cells grown on 96-well white culture plate at the density of 4 × 10^3^ cells/well were treated with each inhibitor for 72 h unless otherwise indicated. Cell viability was quantified using Cell Titer-Glo assay kit (Promega, Madison, Wisconsin), and the results were expressed as a ratio relative to that of the control cells treated with DMSO.

### Western blot analysis

The cells with or without the treatment were subjected to Western blot analysis as described [21] with minor modifications. Cells were lysed in lysis buffer [20 mM Tris–HCl (pH 7.5), 150 mM NaCl, 1 mM EDTA, 1 % Triton X-100, 20 mM NaF, 1 mM Na_3_VO_4_, 0.1 % protease inhibitor cocktail (Sigma-Aldrich, St. Louis, Missouri)]. Protein concentrations of cell lysates were measured with DC protein assay kit (Bio-Rad, Hercules, California), and equal amounts of the protein samples for each experiment (1–20 μg) were separated by SDS–PAGE and transferred to Immobilon P (Millipore). Blots were incubated with respective primary and secondary antibodies at room temperature for 1 h. Proteins were detected using chemiluminescent substrate (Pierce, Rockford, Illinois) according to the manufacturer’s protocol and either LAS-4000 (Fujifilm, Tokyo, Japan) or CEPROS P2 (Fujifilm). The experiments including Aurora kinases were carried out after calibration by using GAPDH as an internal control. Where indicated, the signals were semi-quantitated by using Image J [22].

### Clustering analysis of drug inhibition pattern

To find the target molecules responsible for the anti-proliferative effect of midostaurin in breast cancer, a hierarchal clustering method based on the drug efficacy value was applied with minor modifications [13, 23]. Briefly, the GI50 values, the concentrations needed for each of 74 drugs with known target(s) to inhibit proliferation by 50 % of 45 breast cancer cell lines [14], were transformed using logarithm function to approximate normal distribution. The viability of cell lines in the presence of midostaurin, also having near normal distribution, was then merged to the GI50 value dataset. Subsequently, the cell lines were excluded, which miss the values of more than half of the inhibitors. All of the resting values were transformed to z-score in each drug for the normalization, and employed as the dataset in this study. It is worth noting that the normalized GI50 values and the viability data of midostaurin were obtained as relative values, and are thus comparable among the drugs. Pearson’s correlation distance was used for the application of the clustering analysis as a dissimilarity metric of both drugs and cell lines [24]. Since the dataset still contains missing values, the correlation distances were calculated by a pairwise manner. Calculation and visualization of two-dimensional hierarchical clustering were performed with R version 3.0.2 [25, 26].

### *In vitro* kinase assay

GST-tagged Aurora kinase A and Aurora kinase B were purchased from Carna Biosciences (Kobe, Japan). The kinase activity of Aurora kinases A and B in the presence of either midostaurin or VX-680 was evaluated using Aurora Family Kinase Assay Kit (CycLex, Nagano, Japan) according to manufacturer’s protocol.

### Immunocytochemistry

Cells grown on coverslip were arrested by a single thymidine block with 2 mM thymidine for 24 h as described [27] with minor modifications, and subsequently cultured in the thymidine-free medium in the presence or absence of each inhibitor for 14 h. Resultant cells were fixed with methanol for 3 min at −20 °C. Blocking and incubation with antibodies were performed at room temperature in phosphate-buffered saline containing 0.05 % Tween 20 and 4 % bovine serum albumin. Cells were counterstained with Hoechst 33342 (0.5 μg/ml), mounted using FluoroSave reagent (Calbiochem, Darmstadt, Germany), and observed under BZ-9000 (Keyence, Japan).

### Cell cycle analysis

Cells were cultured with each inhibitor for various periods, harvested with trypsin, and fixed with 70 % ethanol at −20 °C overnight. Thereafter, the cells were incubated in phosphate-buffered saline containing 0.25 mg/ml DNase-free RNase (Nippon Gene, Tokyo, Japan) at 37 °C for 15 min. Subsequently, an equal volume of propidium iodide solution (50 μg/ml) was added. Samples were analyzed with FACS Verse (BD Biosciences, San Jose, California).

### Statistical analysis

Statistical analyses were performed with R version 3.0.2 [25, 26]. Numbers of the experiments, standard deviations (s.d.), and p-values were indicated in each experiment.

## Results

### Anti-proliferative effect of midostaurin on breast cancer cell lines

**Fig. 1:**
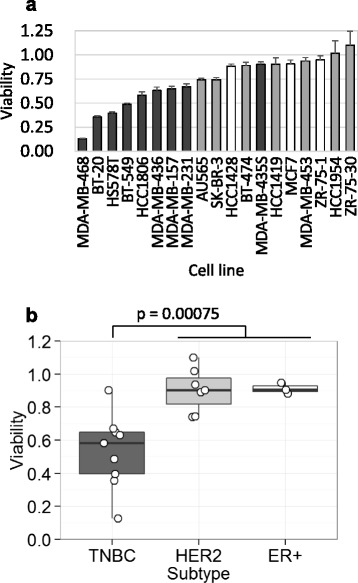
Growth inhibition of breast cancer cell lines by midostaurin. The 19 breast cancer cell lines were treated with 1 μM midostaurin for 72 h, and cell viability was measured. **a** Cell viability shown as a ratio relative to the control sample without treatment. Bars are 1 s.d. of quintuple experiments. Breast cancer subtypes are indicated as follows: gray, TNBC; light gray, HER2; white, ER+. **b** Box plot showing relative viability according to breast cancer subtypes. TNBC vs. non-TNBC, *p* = 0.00075 by Welch’s t-test

**Fig. 2:**
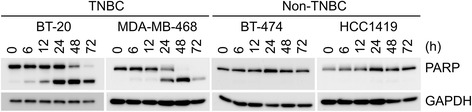
Apoptosis induction by midostaurin. TNBC and non-TNBC cells indicated were treated with 1 μM midostaurin for various periods, and then subjected to Western blot analysis using the antibody against PARP to detect its cleavage. GAPDH was employed as a control

A panel of 19 cell lines, representing three subtypes of human breast cancer, 3 of ER+, 7 of HER2, and 9 of TNBC, were treated with different concentrations of midostaurin, and cell viability was measured (Additional file 2). The effect of midostaurin differed among the cell lines, and thus the viability was compared at 1 μM (Fig. 1a), because the plasma concentrations of the drug in clinical trial for AML have been reported to be a few μM [9]. The TNBC cells except for one line were more sensitive to midostaurin than non-TNBC subtypes such as ER+ and HER2 cells (Fig. 1a): the mean viability values of TNBC and non-TNBC cell lines were 0.53 and 0.91, respectively. The difference between TNBC and non-TNBC subtypes was shown by box plot and was statistically significant (Fig. 1b). The effect of midostaurin on cell death was examined by measuring the cleavage of PARP, as a marker of apoptosis (Fig. 2). In consistent with the result of cell viability, midostaurin brought the cleavage of PARP in TNBC cell lines, BT-20 and MDA-MB-468, but the fragment was not detected in non-TNBC cell lines, BT-474 and HCC1419. These results indicate that midostaurin induces apoptosis preferentially in TNBC cells. Midostaurin was initially generated as a PKC inhibitor [6], and the expression level of the PKC isoforms was evaluated in the breast cancer cell lines by Western blot analysis. PKC isoforms were detected in the breast cancer cell lines such as PKC-α and PKC-βII of the conventional PKC group as well as PKC-δ and PKC-ε of the novel PKC group (Additional file 3). Midostaurin suppressed the PKC-mediated protein phosphorylation as judged by Western blot analysis using the p-Serine PKC substrates antibody in MDA-MB-468 cell line (Additional file 4). The correlation of the expression level of the PKC isoforms with the TNBC cell lines was, however, not observed. On the other hand, it is well known that TNBC cancer cells frequently express EGF receptor although other two subtypes do not [28]. Therefore, the effect of midostaurin was examined on the phosphorylation of EGF receptor and its downstream EGF signaling mechanisms including Akt and Erk kinases. While the treatment of midostaurin at 1 μM induced apoptosis by 24 h as judged by the cleavage of PARP, no significant suppression of the phosphorylation of EGFR (p-EGFR Tyr1068), GSK-3β (p-GSK-3β Ser9), and Erk (p-Erk Thr202/Thr204) was observed during the period (Additional file 4). In addition, lapatinib, a potent inhibitor of the EGF receptor kinase, did not suppress viability of MDA-MB-468 cells, as described previously [29], or enhance the effect of midostaurin (data not shown). Namely, these observations indicate that midostaurin does not target EGF receptor in the TNBC cells.

### Clustering analysis of inhibitors

**Fig. 3:**
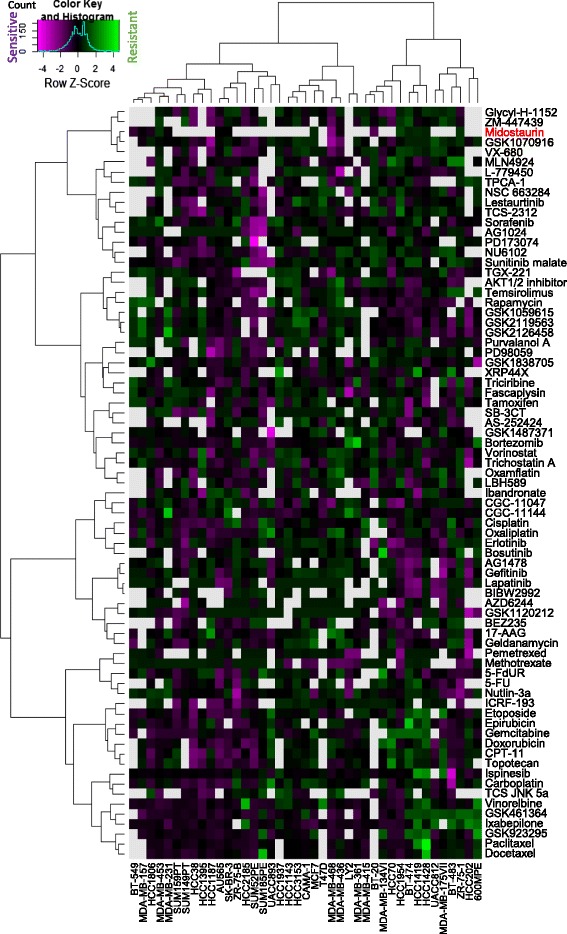
Hierarchical clustering of drugs based on similarity of anti-proliferation effect profiles on breast cancer cell lines. Two-dimensional hierarchical clustering was applied to growth inhibitory activity data of 76 anticancer drugs, including midostaurin, of a panel of breast cancer cell lines. The sensitivity level against each drug in a single cell line relative to its mean value across all cell lines was depicted according to the color scale shown at the upper left corner in the figure, and gray indicates missing values. Drugs with similar mechanisms of action tended to cluster together

To find the target molecule(s) of midostaurin responsible for its anti-proliferative effect, the drug efficacy pattern of midostaurin against the panel of cancer cell lines was compared with that of the drugs having known targets by clustering analysis. The dataset of drug sensitivity of 45 breast cancer cell lines against various known target [14] were utilized as the reference data. The reference data lack HS578T and MDA-MB-435S and contain 17 of the 19 cell lines employed in this study, and were merged with the drug sensitivity data of midostaurin of 17 cell lines, while ZR-75-30 in Fig. 1a and other three cell lines in the reference data were excluded because of excess missing values. The resulting data of 75 drugs, 74 drugs in the reference data and midostaurin, and 41 cell lines were then applied for clustering analysis as shown in Fig. 3. Gefitinib and AG1478, for example, which are potent inhibitors of the EGF receptor kinase [30], were classified into the same cluster (Lines No. 46 and 47 from the top of the panel, respectively). Doxorubicin, CPT-11, and topotecan, which are potent inhibitors of the topoisomerases [14] were classified into the same cluster (Lines No. 64 to 66 from the top of the panel, respectively). These data are similar to the previous report [14] confirming the validity of the clustering analysis. Under these conditions, midostaurin was found in the cluster with VX-680 and GSK1070916, Aurora kinase inhibitors [14, 31]. These results suggest that Aurora kinase is a candidate for the target, which is responsible for the anti-proliferative effect of midostaurin in the TNBC cell lines.

### Effect of midostaurin on Aurora kinases

**Fig. 4:**
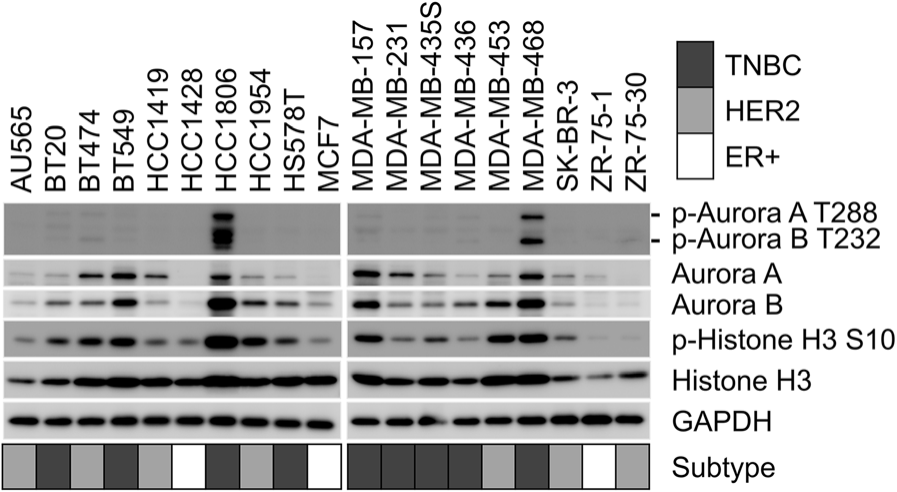
Western blot analysis of breast cancer cell lines. Cell lysates from the 19 breast cancer cell lines were subjected to Western blot analysis. Aurora kinases A and B, p-Aurora kinase A and B, and Histone H3 and p-Histone H3 Ser10 were detected using respective antibodies. The experiments were carried out after calibration by using GAPDH as an internal control. Breast cancer subtypes are indicated as follows: gray, TNBC; light gray, HER2; white, ER+

**Fig. 5:**
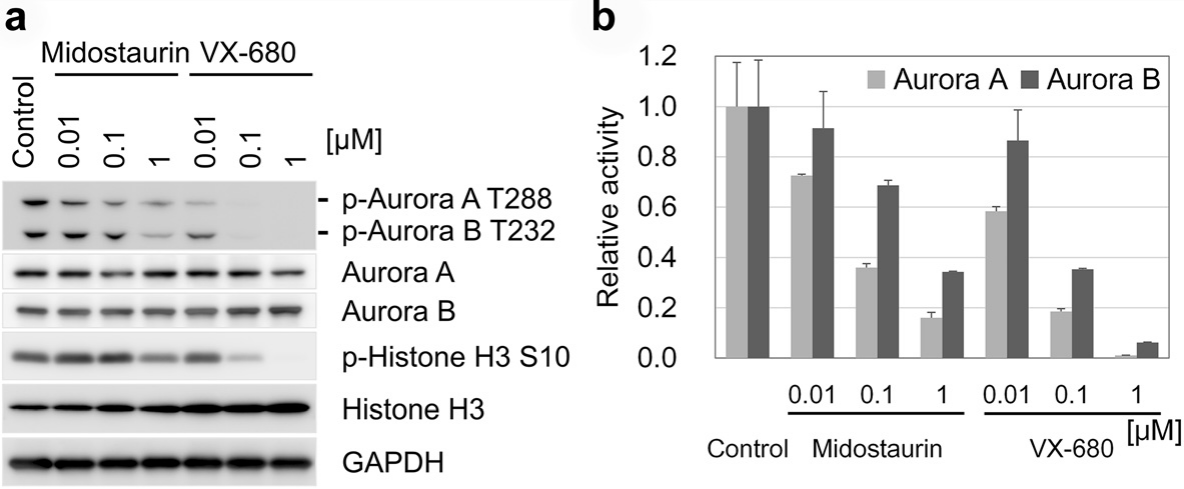
Inhibition of Aurora kinases A and B by midostaurin. The effect of midostaurin on Aurora kinases A and B was examined comparing with that of VX-680. **a** MDA-MB-468 cells were treated with midostaurin or VX-680 at indicated concentrations for 3 h, and then subjected to Western blot analysis using the antibodies as in Fig. 4. **b** *In vitro* kinase activity of Aurora kinases A and B was measured in the presence of midostaurin or VX-680 at indicated concentrations, and the results were shown as a ratio relative to the control sample in the absence of the inhibitor. Bars are 1 s.d. of duplicate experiments

**Fig. 6:**
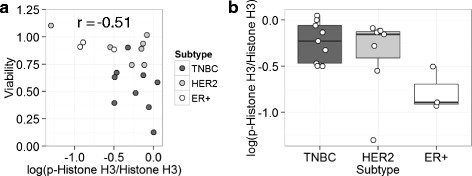
Association between phosphorylation status of Histone H3 Ser10 and viability of midostaurin treated cells in breast cancer cell lines. The phosphorylation of Histone H3 Ser10 and Histone H3 in breast cancer cell lines was semi-quantified by densitometry after Western blot analysis. The phosphorylation level is determined as a ratio of the value of the phosphorylation at Ser10 against that of the total protein of Histone H3 and indicated as p-Histone H3/Histone H3. The values are shown in as relative to MDA-MB-468 cell. **a** The association of the phosphorylation level of Histone H3 Ser10 with cell viability after the midostaurin treatment. Pearson’s correlation coefficient: −0.51, *p* = 0.021 by Pearson correlation test. **b** Box plot showing the phosphorylation level of Histone H3 Ser 10 among the breast cancer subtypes

To determine the role of Aurora kinases in TNBC, the expression level of Aurora kinases A and B was examined in the cancer cell lines (Fig. 4). Expression of Aurora kinases A and B was detected among various cell lines, and was generally high in the TNBC cell lines relative to the non-TNBC cell lines. The phosphorylation of Aurora kinases A and B at Thr288 and Thr232, respectively, as well as that of Histone H3 at Ser10 was evaluated an indicator of the Aurora kinase activity [32]. Notably, the phosphorylation of Histone H3 at Ser10, recognized by Aurora kinase B [32], was high in most of the TNBC cell lines in agreement with the expression level of Aurora kinases A and B. The phosphorylation of Aurora kinases A and B was significantly high in two TNBC cell lines, HCC1806 and MDA-MB-468, but was weak in other cell lines. Thus, the effect of midostaurin was examined on these phosphorylation reactions in the TNBC cell line of MDA-MB-468 (Fig. 5a). The midostaurin treatment significantly reduced the Aurora kinase-mediated phosphorylation reactions in the cell line. Although the effect was weaker than that of VX-680, midostaurin at 1 μM attenuated the phosphorylation of Aurora kinases A and B as well as Histone H3. *In vitro* kinase assay showed that midostaurin at 1 μM inhibited the kinase activity of GST-tagged Aurora kinases A and B by 16 and 34 %, respectively (Fig. 5b). Furthermore, the negative correlation was observed between the phosphorylation level of Histone H3 Ser10 and cell viability after the midostaurin treatment (Fig. 6a): Pearson’s correlation coefficient was −0.51 (*p* = 0.021 by Pearson correlation test). Most of the TNBC cell lines showed a high level of phosphorylation of Histone H3 Ser10 with low cell viability. The phosphorylation level of Histone H3 was higher in TNBC cell lines than that in non-TNBC cell lines (Fig. 6b). These results indicate that midostaurin directly inhibits Aurora kinases A and B, and subsequently reduces cell viability predominantly in the TNBC cells.

### Effect of midostaurin on mitotic cells and cell cycle progression

**Fig. 7:**
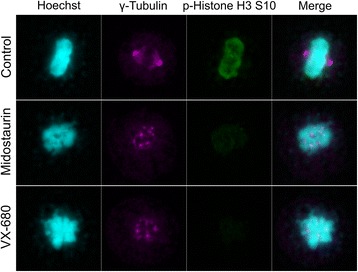
Aberrant mitosis of midostaurin treated cell. MDA-MB-468 cells were synchronized with the thymidine treatment, and subsequently cultured in thymidine-free medium with the indicated inhibitor. Cells were fixed, stained with Hoechst or each antibody, and observed under fluorescence microscope. Nuclear condensation was used as a marker of M phase cells

**Fig. 8:**
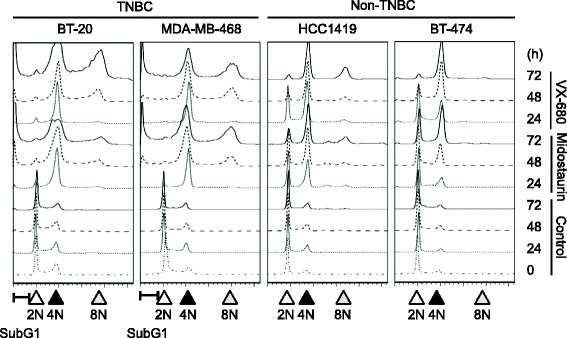
Cell cycle analysis of midostaurin treated cells. TNBC and non-TNBC cells indicated were cultured in the presence and absence of either 1 μM midostaurin or 1 μM VX-680 for indicated periods and subjected to cell cycle analysis. The positions of sub G1, 2N, 4N, and 8N DNA are indicated at the bottom

It has been reported that Aurora kinases are expressed in M phase and the activity is required for the proper mitotic progression [33]. Previous studies have reported that the inhibition of Aurora kinase A suppresses spindle organization [15], and that the inhibition of both Aurora kinases A and B by VX-680 results in the accumulation of 4N DNA cells followed by the induction of 8N DNA cells and apoptosis [32]. To evaluate the effect of midostaurin on mitotic progression, the structure of cell nuclei of the TNBC cell line, MDA-MB-468 was observed after the midostaurin treatment (Fig. 7). Midostaurin, as well as VX-680, suppressed the phosphorylation of Histone H3 Ser10 in M phase cells, and induced defect of spindle organization as judged by immunostaining [34]. Flow cytometric analysis indicated that midostaurin accumulates 4N DNA cells regardless of the breast cancer subtypes suggesting the induction of G2/M arrest (Fig. 8). Furthermore, even 8N DNA cells were found in the TNBC cell lines, BT-20 and MDA-MB-468, and less significantly or not in non-TNBC cell lines. Sub-G1 fraction was observed in the TNBC cell lines but not in non-TNBC cell lines in consistent with the result of the cleavage of PARP (Fig. 2). VX-680, in agreement with the previous study, showed the results almost identical to those of midostaurin [30]. Namely, midostaurin showed the inhibitory effect on cell cycle progression of the cancer cell lines employed as in the case of VX-680, and induced apoptosis restrictively in the TNBC cells.

## Discussion

In this study, it was revealed that midostaurin preferentially suppresses proliferation of TNBC cells among the breast cancer cell lines. Clustering analysis herein predicted that midostaurin shares the target with Aurora kinase inhibitors in the breast cancer cell lines. The Aurora kinase family has three members, Aurora kinases A, B, and C, and the inhibitors identified by the clustering analysis, VX-680 and GSK1070916, are so-called pan-Aurora kinase inhibitors, which suppress all Aurora kinases [31]. Among the Aurora kinase family, Aurora kinases A and B are expressed ubiquitously and have crucial roles for cell division, whereas Aurora kinase C is restricted in testis and is overexpressed in some tumors [15]. Therefore, we concentrated our attention on Aurora kinases A and B in this study. The following analysis indicated that midostaurin shows the properties quite similar to those of VX-680: suppression of the phosphorylation reaction by Aurora kinases in the TNBC cells, inhibition of the *in vitro* kinase reaction of Aurora kinases A and B, and dysregulation of spindle formation, and attenuation of cell cycle progression. Aurora kinase inhibitors are regarded as therapeutic reagents for various cancers [15, 31], and midostaurin is known to inhibit multiple protein kinases including Aurora kinases in comprehensive *in vitro* assay [7]. Midostaurin was, however, shown for the first time to suppress proliferation of the TNBC cells, at least, targeting Aurora kinases. On the other hand, Akt, a serine/threonine protein kinase family, has been proposed to be a target of midostaurin for its anti-cancer effect [35]. It is less possible that Akt is affected by midostaurin because this drug did not attenuate the phosphorylation of GSK-3β by Akt, whereas it immediately inhibited the reaction medicated by PKC as indicated in this study. The results do not necessarily exclude the existence of another target molecule of midostaurin, but it could be concluded that midostaurin acts on the Aurora kinase pathway to inhibit the growth of the TNBC cells.

Aurora kinases A and B play central roles in mitotic process: Aurora kinase A is essential in maturation of centromere and spindle assembly, and Aurora kinase B is required for spindle assembly checkpoint function and in cytokinesis process [15]. VX-680 has been employed as a pan-Aurora kinase inhibitor to analyze the phenotypes by the pharmacological inhibition of both Aurora kinases A and B. Dual inhibition of Aurora kinases A and B induces the phenotypes apparently identical to those of inactivation of Aurora kinase B [36]. Attenuation of Aurora kinase A results in spindle defects, whereas inhibition of Aurora kinase B blocks chromosomal alignment and, moreover, overrides the spindle checkpoint. Therefore, the replication of the nuclear genome occurs without cell division, and the cells show endoreplication such as elevated nuclear content and polyploidy [36]. TNBC is generally considered to be more aggressive with a higher proliferation activity than other breast cancer subtypes. Aurora kinases are activated at M phase of cell cycle for the normal mitotic process, and inhibition of these kinases prevents cell cycle progression and leads to apoptosis [36, 37]. It could be a reason that the TNBC cells have a higher population in the midostaurin-sensitive phase than other breast cancer subtypes. The TNBC cells, on the other hand, frequently express EGF receptor, whereas other two subtypes do not [11]. It has been reported that EGF induces the gene expression of Aurora kinase A through the nuclear translocation of EGF receptor with STAT5 (signal transducer and activator of transcription 5) followed by the recruitment of them to the promoter of Aurora kinase A [38]. It seems thus possible that the TNBC cells contain a high level of Aurora kinase A as one of their malignant properties through the EGF-signaling pathway, and that the resulting overexpression of Aurora kinase A makes the TNBC cells sensitive to midostaurin. On the other hand, overexpression of Aurora kinase C has been found in some cancer cells [15], and thus it is necessary to study the effect of midostaurin on the cancer cells having Aurora kinase C. Recently, it has been reported that VX-680 selectively kills the cells that overexpress Myc [39], and the level of Myc is elevated in TNBC [40]. The precise mechanism of the synthetic lethal interaction between VX-680 and Myc overexpression is not yet known, but midostaurin may also affect the growth the cells overexpressing Myc. Further studies are necessary to elucidate the role of midostaurin in the preferential growth inhibition to develop the drug as an anti-cancer drug against TNBC.

Currently, midostaurin has been evaluated as the drug for the treatment of AML by targeting FLT-3, which is expressed exclusively in hematopoietic cells [12]. The previous clustering analysis of the drug activity data had been carried out using various cancer cells of 60 cell lines [13]. Here, we employed only breast cancer cell lines, in which the effect against FLT-3 was excluded from the analysis, and picked up Aurora kinases as the candidate for the target of midostaurin in TNBC cells. The results obtained in this study suggest that midostaurin shows its anti-cancer effect on AML by targeting not only FLT-3 but also Aurora kinases, especially in leukemia without aberration in FLT-3. This approach employing restricted cancer cell lines may be useful to identify the target molecule(s) of the multi-target protein kinase inhibitors in different cancers.

## Conclusions

Midostaurin has been developed as the drug for the treatment of AML, which has constitutive active FLT-3. The results obtained indicate that midostaurin preferentially inhibits growth of the TNBC cell lines through inhibition of the Aurora kinase family. It is plausible that midostaurin has a potential to be a drug for the treatment of different types of cancer by inhibiting respective target kinases.

## Additional files

Additional file 1:
**Cell culture conditions.** Cell lines employed in this study are summarized with their culture conditions. Additional file 2:
**Growth inhibition of breast cancer cell lines by various concentration of midostaurin.** Cells were treated with various concentrations of midostaurin for 72 h, and cell viability was evaluated and shown as a ratio relative to the control sample without the treatment. Additional file 3:
**PKC isoforms in breast cancer cell lines.** Cell lysates were subjected to Western blot analysis using the antibodies as indicated. PKC-α, PKC-βII, PKC-δ, PKC-ε, and GAPDH were detected. Breast cancer subtypes are indicated as follows: gray, TNBC; light gray, HER2; white, ER+. Additional file 4:
**Signaling cascades in MDA-MB-468 cells.** Cells were cultured in the absence and presence of 1 μM midostaurin for indicated periods, and then subjected to Western blot analysis using the antibodies as indicated. p-EGFR Tyr1068, EGF receptor, p-Akt Ser473, pan-Akt, p-GSK3β Ser9, GSK-3β, p-Erk Thr202/Thr204, Erk 1/2, PARP, p-Serine PKC substrates, and GAPDH were detected.

## Abbreviations

FLT-3: FMS-like tyrosine kinase-3
AML: Acute myeloid leukemia
HER2: Breast cancer subtype expressing human epidermal growth factor receptor 2
ER+: Breast cancer subtype expressing endocrine receptors such as estrogen and progesterone receptors without expression of HER2
TNBC: Triple-negative breast cancer
FBS: Fetal bovine serum
EGF: Epidermal growth factor
PARP: Poly(ADP-ribose) polymerase
GAPDH: Glyceraldehyde-3-phosphate dehydrogenase
DMSO: Dimethyl sulfoxide
