# Supplementary material for: Midostaurin preferentially attenuates proliferation of triple-negative breast cancer cell lines through inhibition of Aurora kinase family
Source: J Biomed Sci. 2015 Jul 4;22(1):48. doi: 10.1186/s12929-015-0150-2 (PMC4491224; doi:10.1186/s12929-015-0150-2)
Supplement: Additional file 1: — Cell culture conditions. Cell lines employed in this study are summarized with their culture conditions. [file 12929_2015_150_MOESM1_ESM.pdf]

Additional file 1

Cell line	ATCC No.	Medium[a]	FBS	Addition	CO_2
AU565	CRL-2351	RPMI 1640	10%		5%
BT-20	HTB-19	MEM	10%		5%
BT-474	HTB-20	DMEM	10%		5%
BT-549	HTB-122	RPMI 1640	10%	1 µg/ml bovine insulin	5%
HCC1419	CRL-2326	RPMI 1640	10%		5%
HCC1428	CRL-2327	RPMI 1640	10%		5%
HCC1806	CRL-2335	RPMI 1640	10%		5%
HCC1954	CRL-2338	RPMI 1640	10%		5%
HS578T	HTB-126	DMEM	10%	10 µg/ml bovine insulin	5%
MCF7	HTB-22	MEM[b]	10%	10 µg/ml bovine insulin	5%
MDA-MB-157	HTB-24	Leibovitz's L-15	10%		0%
MDA-MB-231	HTB-26	Leibovitz's L-15	10%		0%
MDA-MB-435S	HTB-129	Leibovitz's L-15	10%	10 µg/ml bovine insulin	0%
MDA-MB-436	HTB-130	Leibovitz's L-15	10%	10 µg/ml bovine insulin, 10 µg/ml glutathione	0%
MDA-MB-453	HTB-131	Leibovitz's L-15	10%		0%
MDA-MB-468	HTB-132	Leibovitz's L-15	10%		0%
SK-BR-3	HTB-30	McCoy's 5a	10%		5%
ZR-75-1	CRL-1500	RPMI 1640	10%		5%
ZR-75-30	CRL-1504	RPMI 1640	10%		5%

a. Manufacturers of basal medium are Sigma-Aldrich (St. Louis, Missouri) except for MEM for MCF7.

b. MEM from Life Technologies (Carlsbad, California)
