# Supplementary figures and images for: Midostaurin preferentially attenuates proliferation of triple-negative breast cancer cell lines through inhibition of Aurora kinase family
Source: J Biomed Sci. 2015 Jul 4;22(1):48. doi: 10.1186/s12929-015-0150-2 (PMC4491224; doi:10.1186/s12929-015-0150-2)

## Additional file 2

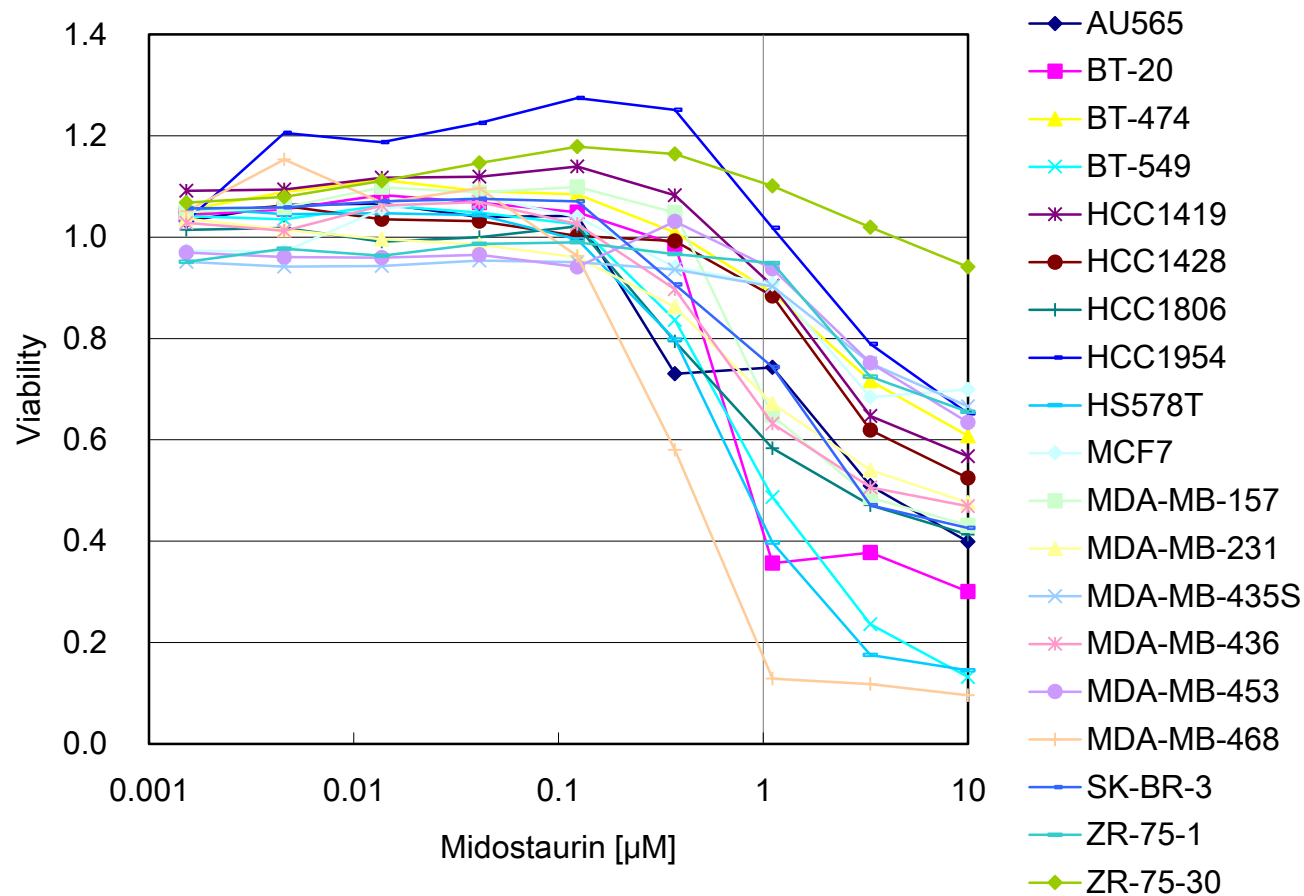

Supplement: Additional file 2: — Growth inhibition of breast cancer cell lines by various concentration of midostaurin. Cells were treated with various concentrations of midostaurin for 72 h, and cell viability was evaluated and shown as a ratio relative to the control sample without the treatment. [file 12929_2015_150_MOESM2_ESM.pdf]

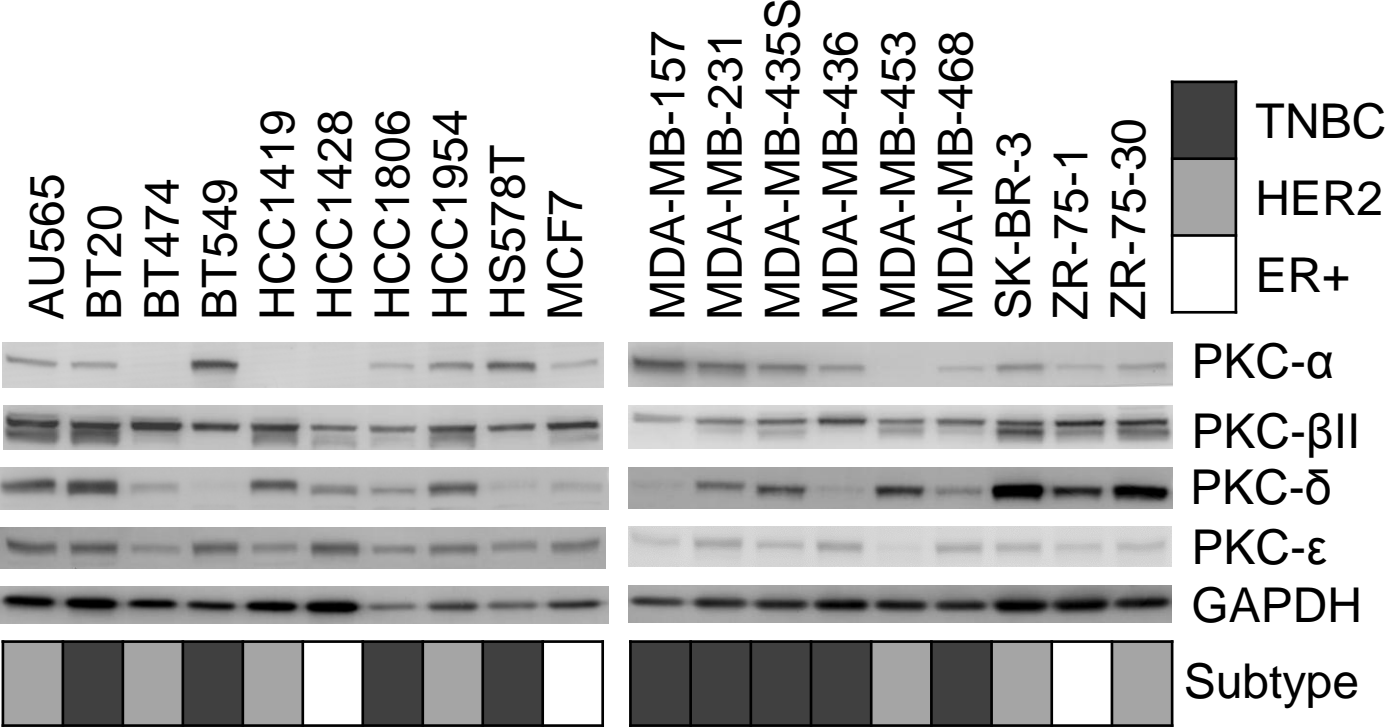

Supplement: Additional file 3: — PKC isoforms in breast cancer cell lines. Cell lysates were subjected to Western blot analysis using the antibodies as indicated. PKC-α, PKC-βII, PKC-δ, PKC-ε, and GAPDH were detected. Breast cancer subtypes are indicated as follows: gray, TNBC; light gray, HER2; white, ER+. [file 12929_2015_150_MOESM3_ESM.pdf]

Additional file 4

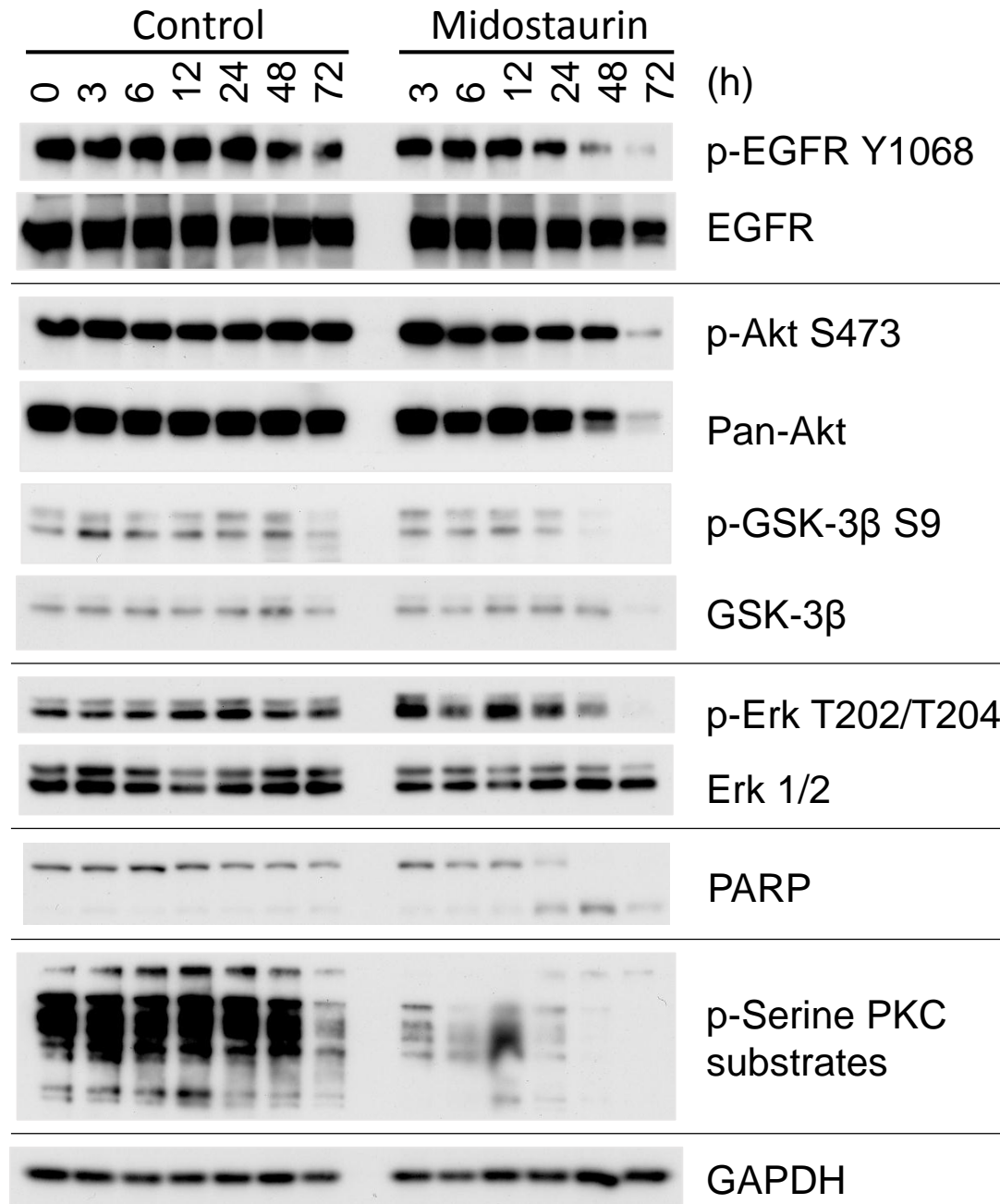

Supplement: Additional file 4: — Signaling cascades in MDA-MB-468 cells. Cells were cultured in the absence and presence of 1 μM midostaurin for indicated periods, and then subjected to Western blot analysis using the antibodies as indicated. p-EGFR Tyr1068, EGF receptor, p-Akt Ser473, pan-Akt, p-GSK3β Ser9, GSK-3β, p-Erk Thr202/Thr204, Erk 1/2, PARP, p-Serine PKC substrates, and GAPDH were detected. [file 12929_2015_150_MOESM4_ESM.pdf]
